# KIBRA upregulation increases susceptibility to podocyte injury and glomerular disease progression

**DOI:** 10.1172/jci.insight.165002

**Published:** 2023-04-10

**Authors:** Kristin Meliambro, Yanfeng Yang, Marina de Cos, Estefania Rodriguez Ballestas, Caroline Malkin, Jonathan Haydak, John R. Lee, Fadi Salem, Laura H. Mariani, Ronald E. Gordon, John M. Basgen, Huei Hsun Wen, Jia Fu, Evren U. Azeloglu, John Cijiang He, Jenny S. Wong, Kirk N. Campbell

**Affiliations:** 1 Division of Nephrology, Icahn School of Medicine at Mount Sinai, New York, New York, USA.; 2 Division of Nephrology and Hypertension, Department of Medicine, Weill Cornell Medicine, New York, New York, USA.; 3 Department of Laboratory Medicine and Pathology, Mayo Clinic, Jacksonville, Florida, USA.; 4 Division of Nephrology, Department of Medicine, University of Michigan, Ann Arbor, Michigan, USA.; 5 Department of Pathology, Icahn School of Medicine at Mount Sinai, New York, New York, USA.; 6 Stereology and Morphometry Laboratory, Charles R. Drew University of Medicine and Science, Los Angeles, California, USA.; 7 Department of Pharmacological Sciences, Icahn School of Medicine at Mount Sinai, New York, New York, USA.

**Keywords:** Cell Biology, Nephrology, Cell migration/adhesion, Chronic kidney disease

## Abstract

Despite recent progress in the identification of mediators of podocyte injury, mechanisms underlying podocyte loss remain poorly understood, and cell-specific therapy is lacking. We previously reported that kidney and brain expressed protein (KIBRA), encoded by *WWC1*, promotes podocyte injury in vitro through activation of the Hippo signaling pathway. KIBRA expression is increased in the glomeruli of patients with focal segmental glomerulosclerosis, and KIBRA depletion in vivo is protective against acute podocyte injury. Here, we tested the consequences of transgenic podocyte-specific *WWC1* expression in immortalized human podocytes and in mice, and we explored the association between glomerular *WWC1* expression and glomerular disease progression. We found that KIBRA overexpression in immortalized human podocytes promoted cytoplasmic localization of Yes-associated protein (YAP), induced actin cytoskeletal reorganization, and altered focal adhesion expression and morphology. *WWC1*-transgenic (KIBRA-overexpressing) mice were more susceptible to acute and chronic glomerular injury, with evidence of YAP inhibition in vivo. Of clinical relevance, glomerular *WWC1* expression negatively correlated with renal survival among patients with primary glomerular diseases. These findings highlight the importance of KIBRA/YAP signaling to the regulation of podocyte structural integrity and identify KIBRA-mediated injury as a potential target for podocyte-specific therapy in glomerular disease.

## Graphical abstract

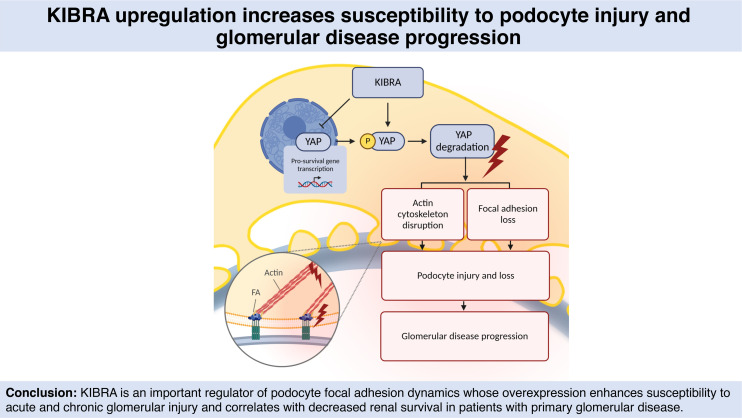

## Introduction

Podocyte injury and reduction in podocyte number are key events leading to the development and progression of glomerular diseases. Though mechanisms underlying podocyte loss have been identified in human disease and experimental animal models, the critical signaling pathways regulating podocyte structural integrity and podocyte–glomerular basement membrane (GBM) attachment remain areas of active investigation (1–4). Despite advances in recent years, therapeutic targets are still scarce, and the deficiency of cell-specific therapies for the treatment of glomerular diseases remains.

Kidney and brain expressed protein (KIBRA, also known as WW and C2 containing 1 protein and encoded by the *WWC1* gene), is a cytoplasmic scaffold protein that was initially identified as a putative binding partner for the pro-injury dual-compartment molecule dendrin (5, 6). In the brain, interactions with dendrin and PKMζ, a brain-specific isoform of PKCζ, likely contribute to KIBRA’s regulation of memory performance (7). Single-nucleotide polymorphisms (SNPs) in *WWC1* have also been associated with an increased risk of late-onset Alzheimer disease (8). In the kidney, early studies in podocytes identified KIBRA as a binding partner of the actin-associated protein synaptopodin and the polarity proteins PATJ (9) and PKCζ (10), suggesting a role for KIBRA in the regulation of podocyte motility.

Subsequently, KIBRA was found to be a key upstream regulator of the Hippo signaling pathway, a conserved kinase cascade from *Drosophila* to mammals that controls organ size through inhibition of cell proliferation and promotion of apoptosis (11, 12). Importantly, dysregulation of the Hippo pathway contributes to tumor development. KIBRA acts in conjunction with proteins Merlin and Expanded (Ex) in *Drosophila* and NF2 and Willin/Ex1/FRMD6 in mammals to regulate Hippo signaling via interactions with core pathway kinases, including MST1/2 and LATS1/2 (13–15). Specifically, KIBRA promotes the phosphorylation of LATS1/2, driving the downstream phosphorylation and cytoplasmic redistribution of Yorkie in *Drosophila* and of Yes-associated protein (YAP) in mammalian cells (16, 17). Since YAP is a transcriptional coactivator that acts in concert with the TEAD family of transcription factors to promote cell survival, growth, and differentiation (18), KIBRA’s demonstrated antagonism of YAP signaling has pathophysiologic relevance to the fields of oncology and nephrology. The identification of KIBRA as a tumor suppressor protein and YAP as an oncogene has led to studies linking the expression of KIBRA and various interacting partners with clinical prognosis in breast cancer and gastric cancer, among other malignancies (19). In the kidney, we previously demonstrated that KIBRA/YAP signaling is important to the regulation of podocyte actin cytoskeleton dynamics (20). KIBRA glomerular expression is also associated with human focal segmental glomerulosclerosis (FSGS). Bennett et al. identified *WWC1* as one of the top upregulated genes in a microarray from laser-capture–microdissected FSGS glomeruli (21). Our group demonstrated reduced YAP and increased KIBRA glomerular expression in human FSGS biopsies (20, 22). Furthermore, podocyte-specific YAP deletion in mice resulted in FSGS and renal failure, while constitutive KIBRA-knockout (KIBRA-KO) mice were protected in an acute podocyte injury model. These findings support KIBRA’s role as an important mediator of podocyte injury, yet the direct effects of increased KIBRA expression in vivo and the association between KIBRA expression and glomerular disease outcomes have not yet been defined.

Here we tested the effects of KIBRA overexpression on human podocyte morphology and generated podocyte-specific *WWC1*-transgenic (KIBRA-overexpressing, KIBRA-OE) mice to investigate susceptibility to acute and chronic podocyte injury. We also analyzed longitudinal clinical outcomes associated with *WWC1* expression in primary glomerular disease.

## Results

### KIBRA overexpression in human podocytes alters podocyte focal adhesion dynamics.

**Figure 1:**
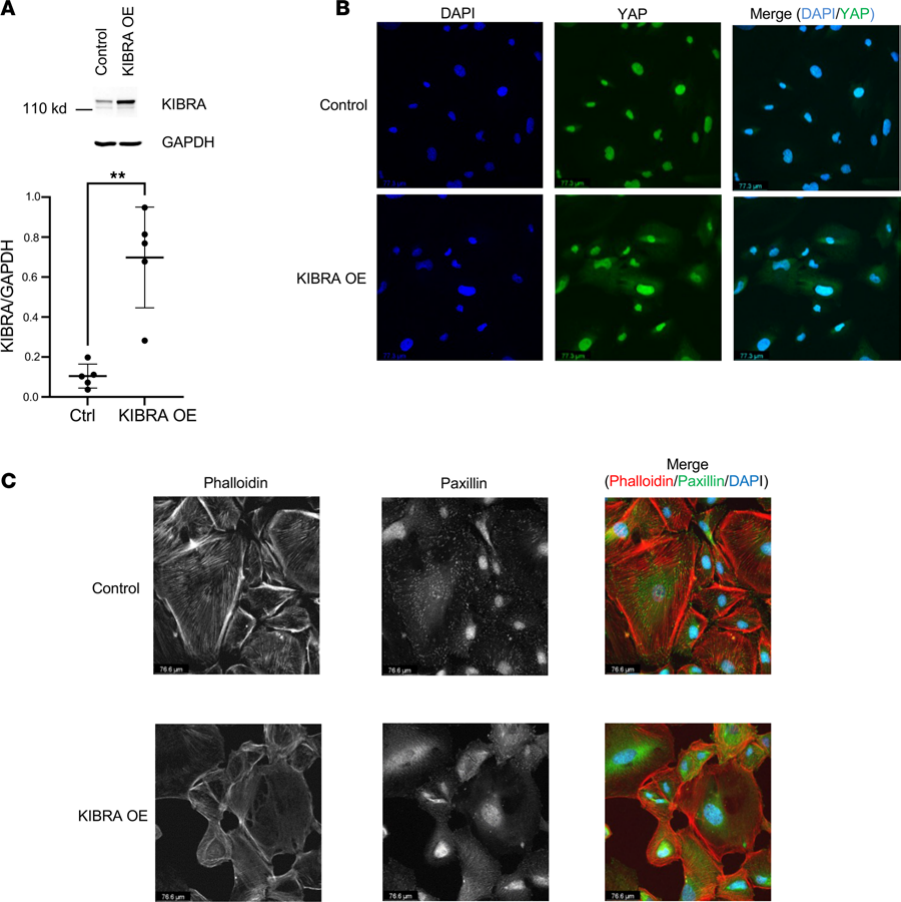
KIBRA overexpression alters YAP localization and disrupts normal podocyte morphology. (**A**) KIBRA overexpression in human podocytes (*n* = 4 experiments). **P < 0.01 by 2-tailed *t* test. Ctrl, control. (**B**) KIBRA-OE podocytes show increased cytoplasmic localization of YAP (green) compared with control podocytes (*n* = 3 experiments). Scale bars: 77.3 μm. (**C**) KIBRA-OE podocytes display disorganization of normal actin fibers, which were also reduced in number (left panel and merge), and dramatic loss of focal adhesions (middle panel and merge), compared with control podocytes (*n* = 3 experiments). Scale bars: 76.6 μm.

**Figure 2:**
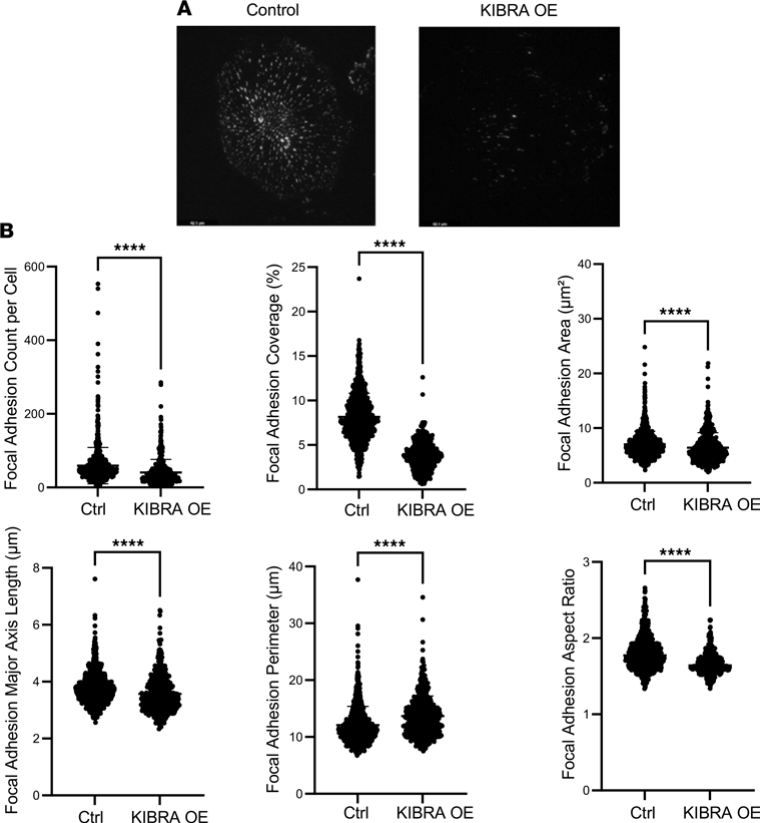
KIBRA overexpression reduces expression and alters parameters of podocyte focal adhesions. (**A**) Representative total internal reflection fluorescence (TIRF) images of control and KIBRA-OE human podocytes imaged with an evanescent field depth of 90 nm. Scale bars: 42.1 μm. (**B**) Quantitative high-content image analysis shows that KIBRA overexpression resulted in a reduction in focal adhesion count and coverage per cell, along with decreased focal adhesion area, major axis length, perimeter, and aspect ratio (*n* = 964 control cells, 485 KIBRA-OE cells). ****P < 0.0001 by 2-tailed Mann-Whitney test. Ctrl, control.

KIBRA was overexpressed via retroviral transduction in human podocytes and increased expression was confirmed via Western blotting, which showed a 7-fold increase in KIBRA protein expression (normalized to GAPDH) in KIBRA-OE podocytes versus control (empty vector) podocytes (Figure 1A). We found that KIBRA-OE podocytes had increased cytoplasmic (inactive) YAP expression with respect to controls (Figure 1B). KIBRA-OE human podocytes appeared dysmorphic, with notable loss of and disorganization of actin stress fibers and reduction in focal adhesion marker expression (Figure 1C). These results are consistent with our previous findings in mouse podocytes (20). To further characterize KIBRA-induced changes in focal adhesion parameters, we performed high-content image analysis of images obtained via total internal reflection fluorescence (TIRF) microscopy (Figure 2A). Using a machine learning algorithm, we found that KIBRA-OE podocytes displayed significant reduction in focal adhesion count per cell, coverage, area, major axis length, perimeter, and aspect ratio (Figure 2B). Taken together, these results suggest that KIBRA overexpression in vitro yielded structural changes that could correlate with podocyte detachment and loss in vivo. Interestingly, despite the severity of the KIBRA-induced disruption of actin fibers and focal adhesions in human podocytes in culture, apoptosis levels, as assessed by Western blotting for cleaved caspase-3, were undetectable in both control and KIBRA-OE podocytes at baseline (Supplemental Figure 1A; supplemental material available online with this article; https://doi.org/10.1172/jci.insight.165002DS1). However, following a 24-hour treatment period with Adriamycin (ADR; 0.25 μg/mL), cleaved caspase-3 levels increased in KIBRA-OE podocytes to a greater extent than in controls.

### KIBRA overexpression in vivo increases susceptibility to acute podocyte injury.

**Figure 3:**
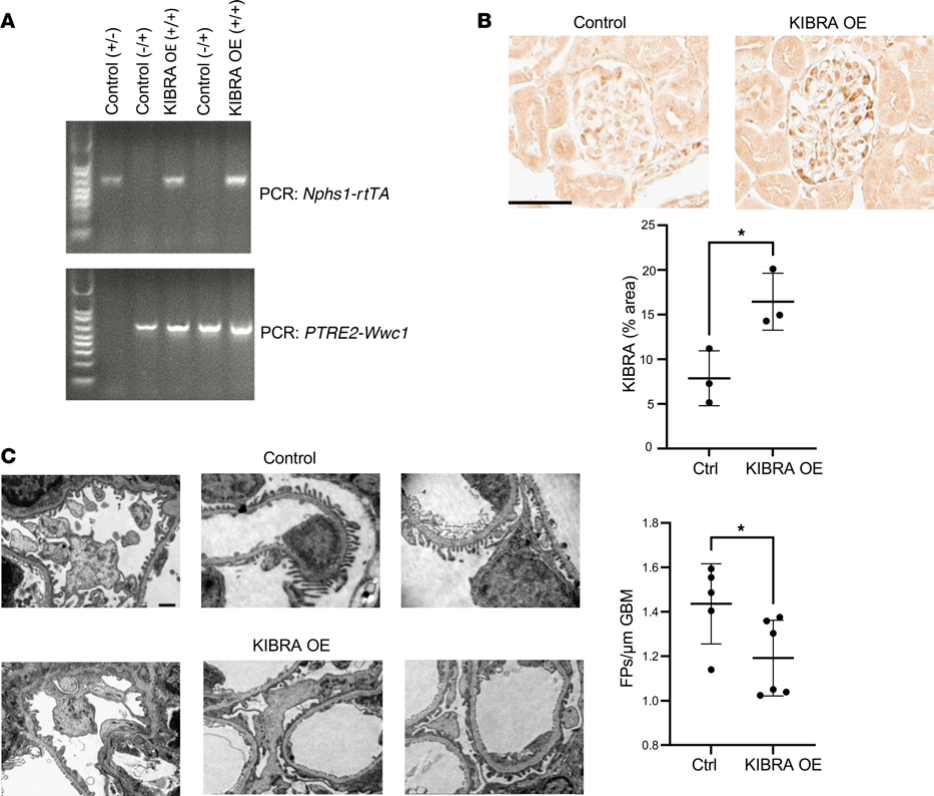
Podocyte-specific overexpression of KIBRA increases susceptibility to acute glomerular injury. (**A**) Representative PCR genotyping of single transgenic (lanes 1, 2, and 4) and double transgenic KIBRA-OE (lanes 3 and 5) mice (*n* = 3 control, 2 KIBRA-OE mice). (**B**) Podocyte-specific KIBRA overexpression was confirmed by IHC staining after Dox induction (*n* = 3 control, 3 KIBRA-OE mice). Scale bar: 50 μm. *P < 0.05 by 2-tailed *t* test. (**C**) Representative TEM images demonstrate significant foot process (FP) effacement in KIBRA-OE mice after protamine sulfate perfusion, confirmed by quantification of average FP number per length of GBM (*n* = 5 control, 6 KIBRA-OE mice). Original magnification, ×10,000. Scale bar: 50 μm. *P < 0.05 by 2-tailed Mann-Whitney test.

Given our previously published findings that constitutive deletion of KIBRA in mice was protective against acute podocyte injury (20) and our in vitro demonstrations of KIBRA-induced structural derangements, we next hypothesized that KIBRA overexpression in vivo would either directly lead to a glomerular disease phenotype or would enhance susceptibility to acute and chronic glomerular injury. To investigate this, we generated doxycycline-inducible (Dox-inducible) podocyte-specific KIBRA-OE mice by injecting a construct containing a tetracycline-dependent promoter upstream of *WWC1* (*TRE-WWC1*) into wild-type FVB/N oocytes and then breeding the subsequent offspring with an FVB/N transgenic line of mice containing the podocyte-specific nephrin reverse tetracycline-controlled transactivator (*Nphs1-rtTA*) (Figure 3A). SNP analysis confirmed the FVB/N background. Double-transgenic (*Nphs1-rtTA^+^* *TRE-WWC1^+^*) KIBRA-OE mice were screened for Dox responsiveness at 10.5 weeks of age after receiving Dox-supplemented chow for 1 week. Podocyte-specific KIBRA overexpression was confirmed via quantification of immunohistochemistry (IHC) staining of glomeruli in kidney cortex sections following transcardiac paraformaldehyde (PFA) perfusion and organ harvesting (Figure 3B), given previous findings of KIBRA expression limited to podocytes in rat glomeruli (9), as well as our prior demonstration of KIBRA colocalization with podocyte marker synaptopodin in human glomeruli from renal biopsy specimens (20). Wild-type (*Nphs1-rtTA^–^* *TRE-WWC1^–^*) littermates and single-transgenic (*Nphs1-rtTA^+^* *TRE-WWC1^–^* or *Nphs1-rtTA^–^* *TRE-WWC1^+^*) littermates were used as controls for all subsequent experiments. KIBRA expression was demonstrated to be an average of 2-fold higher in KIBRA-OE mice versus controls based on quantification of IHC staining. Despite KIBRA overexpression in podocytes, a glomerular injury phenotype was not identified in KIBRA-OE mice after Dox supplementation alone. KIBRA-OE mice did not develop albuminuria and they demonstrated normal histology by light microscopy (Supplemental Figure 1B) at the time of sacrifice following 1 week of Dox administration. These findings remained unchanged despite the extension of the period of Dox administration to up to 6 weeks total (data not shown). Therefore, increased KIBRA expression alone was not sufficient to induce glomerular disease in mice.

Given our previous finding that KIBRA-KO mice were protected from protamine sulfate–induced (PS-induced) injury (20)*,* we hypothesized that KIBRA overexpression in vivo could be part of a double-hit mechanism that would accelerate podocyte damage in the setting of underlying glomerular injury. PS is a polycation that can neutralize the negatively charged plasma membrane of podocyte foot processes (FPs), leading to the formation of abnormal tight junctions and reorganization of the actin cytoskeleton, which results in acute FP effacement (23). PS-induced FP effacement is a well-established model of podocyte injury and has been demonstrated to be a calcium-dependent process (24) that is reversible with perfusion of the polyanion heparin (25). Following 2 weeks of Dox-supplemented chow*,* 12-week-old KIBRA-OE and control mice were perfused with PS. Transmission electron microscopy (TEM) imaging of kidney sections from both groups revealed that KIBRA-OE mice had more severe and contiguous FP effacement than did control littermates (*n* = 5 control mice and 6 KIBRA-OE mice) (Figure 3C). Female and male KIBRA-OE mice (*n* = 3 female and 3 male mice) sustained similar degrees of PS-induced FP effacement, indicating comparable susceptibility to acute podocyte injury. These findings show that KIBRA increased susceptibility to acute podocyte injury in vivo.

### KIBRA overexpression increases glomerular injury in ADR nephropathy.

**Figure 4:**
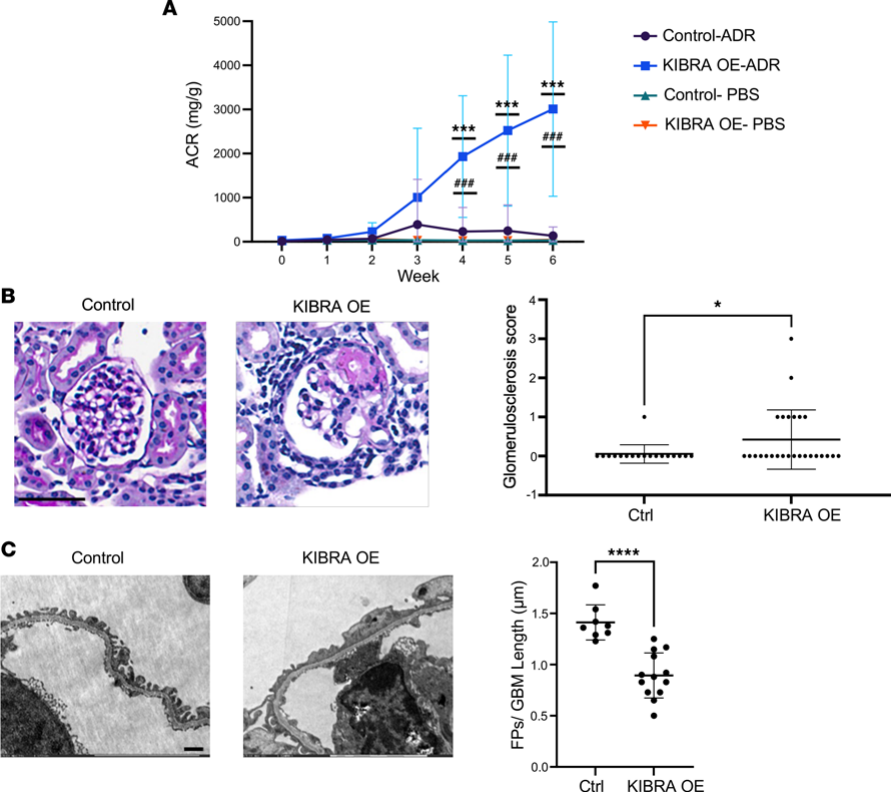
Podocyte-specific overexpression of KIBRA increases podocyte injury in Adriamycin-nephropathy in mice. KIBRA-OE (*n* = 13) and control mice (*n* = 9) were given Dox-supplemented chow for 1 week before ADR (ADR) or vehicle (PBS) injection and sacrificed 6 weeks after injection. (**A**) Urinary albumin-to-creatinine ratio (ACR) after ADR or PBS injection, where week 0 is the preinjection baseline. ***P < 0.001 for KIBRA OE-ADR versus KIBRA OE-PBS; ^###^*P* < 0.001 for KIBRA OE-ADR versus Control-ADR; 1-way ANOVA with Tukey’s multiple-comparison test. (**B**) Representative images of PAS-stained kidneys (original magnification, ×40; scale bar: 50 μm) and glomerulosclerosis scores (segmental + global glomerulosclerosis) per mouse. *P < 0.05 by 2-tailed *t* test. (**C**) Representative TEM images (original magnification, ×10,000; scale bar: 50 μm) and quantification of average foot process (FP) number per GBM length. ****P < 0.0001 by 2-tailed *t* test. Ctrl, control.

We next tested the effect of KIBRA overexpression in the chronic glomerular disease model, ADR-induced nephropathy. In rodents, ADR administration provokes albuminuria with a glomerular injury pattern that is histologically similar to human FSGS, with podocyte FP effacement, focal segmental and global glomerular sclerosis, and interstitial inflammation and fibrosis (26). We selected this well-established model of chronic glomerular injury due to its reproducibility, reliability, and low risk of mortality and adverse events (mainly weight loss) (27, 28). Dox-supplemented chow was given for 1 week to 9.5-week-old KIBRA-OE and control littermates, after which time ADR was administered (15 mg/kg) via retro-orbital injections. A separate group of 9.5-week-old KIBRA-OE and control mice that received Dox-supplemented chow for 1 week followed by retro-orbital PBS injections served as experimental controls. Weekly urine collections were obtained for 6 weeks, and albuminuria was quantified via albumin ELISA and urine creatinine assays. These mice were continuously fed Dox-supplemented chow to ensure continued KIBRA overexpression until the time of sacrifice at 6 weeks after ADR/PBS. Following ADR injection, substantial albuminuria developed in KIBRA-OE mice by the 3-week time point (mean albumin-to-creatinine ratio [ACR] 1004.4 mg/g) and continued to increase up until the time of sacrifice at 6 weeks after ADR (mean ACR 3008.7 mg/g). By contrast, control mice had significantly less albuminuria that peaked at the 3-week time point (mean ACR 390.4 mg/g) and diminished by the 6-week time point (134.8 mg/g) (Figure 4A). There were no sex-based differences observed in susceptibility to ADR-induced injury, as female and male KIBRA-OE mice had similar amounts of proteinuria at the 6-week time point (*n* = 6 female and 7 male mice). Histological analysis of kidney sections from mice that were sacrificed and perfused 6 weeks after ADR revealed segmental and global glomerular sclerosis in 6 out of 13 KIBRA-OE mice, while only 1 out 9 control mice demonstrated segmental sclerosis, and the remainder of the control mice had normal-appearing glomeruli (Figure 4B). Further ultrastructural analysis via TEM revealed extensive FP effacement in KIBRA-OE mice, while control mice had significantly less and patchier FP effacement (Figure 4C).

**Figure 5:**
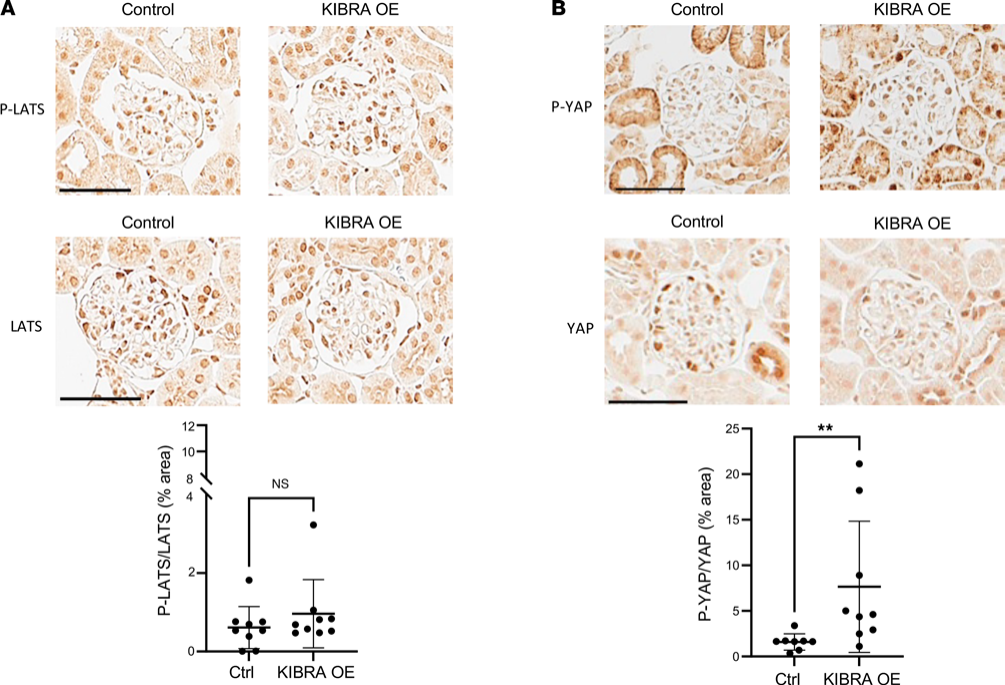
LATS and YAP phosphorylation in ADR-treated KIBRA-OE mice. Adriamcyin-treated KIBRA-OE mice show (**A**) a nonsignificant increase in p-LATS/LATS expression compared with control mice (*n* = 9 control, 9 KIBRA-OE mice) and (**B**) significantly increased p-YAP/YAP expression compared with control mice (*n* = 8 control, 9 KIBRA-OE mice). Original magnification, ×40. Scale bars: 50 μm. **P < 0.01 by 2-tailed Mann-Whitney test. NS, not significant.

**Figure 6:**
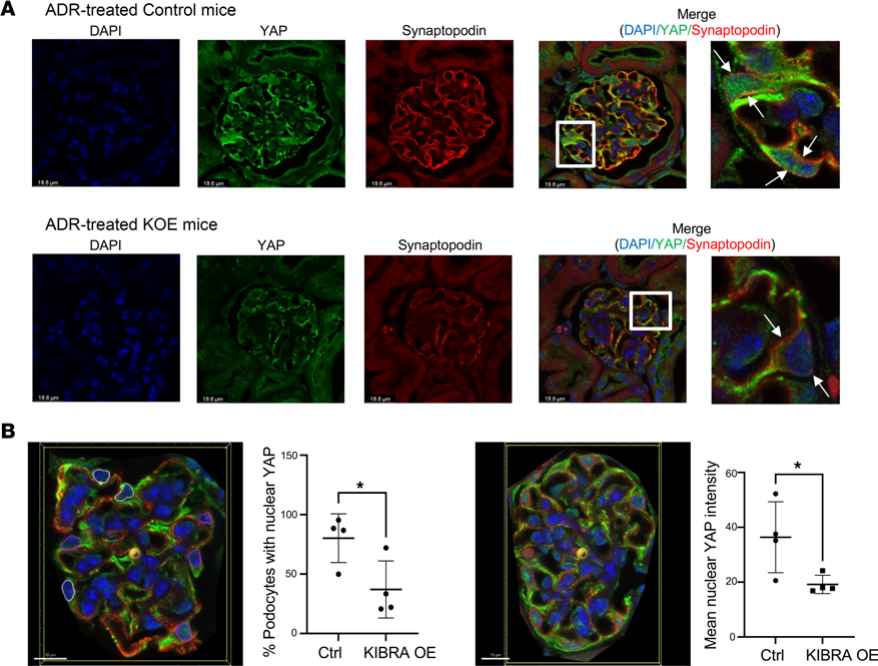
YAP localization in ADR-treated mice. (**A**) ADR-treated KIBRA-OE mice show decreased nuclear YAP and increased cytoplasmic YAP localization in podocytes (podocytes in zoomed areas labeled by white arrows) compared with ADR-treated control mice. Original magnification, ×63. Scale bars: 19.6 μm and 5.5 μm (zoomed areas labeled by white boxes). (**B**) Representative images illustrating 2 methods utilized to quantify nuclear YAP localization: quantification of percentage of podocytes with positive nuclear YAP staining based on a set threshold intensity within DAPI-labeled podocyte nuclei (left image: pink = podocyte nuclei positive for YAP staining, white = podocyte nuclei negative for YAP staining), and quantification of mean nuclear YAP intensity within DAPI-labeled podocyte nuclei (right image: red = all podocyte nuclei). Scale bars: 10 μm. *n* = 4 control, 4 KIBRA-OE mice.*P < 0.05 by 2-tailed *t* test.

**Figure 7:**
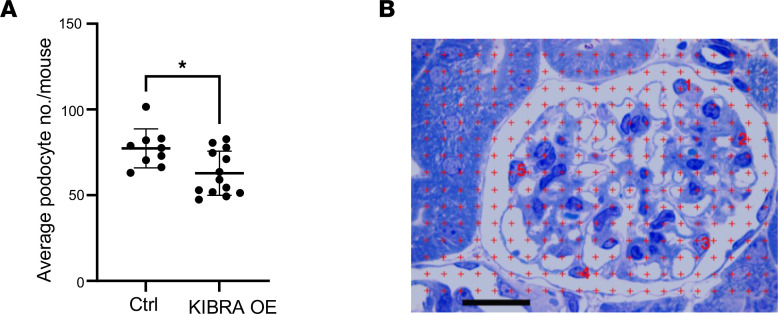
Podocyte number in ADR-treated mice. (**A**) ADR-treated KIBRA-OE mice show reduced podocyte number compared with ADR-treated control mice (*n* = 9 control, 13 KIBRA-OE mice). *P < 0.05 by 2-tailed *t* test. (**B**) Glomerulus with superimposed grid illustrating the Weibel-Gomez method utilized to quantify podocyte number per glomerulus. Red numbers identify the podocyte nuclei. Toluidine blue stain. Scale bar: 20 μm.

IHC staining showed that glomeruli of ADR-treated KIBRA-OE mice had a nonsignificant increase in phosphorylated LATS/total LATS (p-LATS/LATS) expression and significantly increased phosphorylated YAP/total YAP (p-YAP/YAP) expression when compared with control mice (Figure 5, A and B). With vehicle (PBS) treatment of KIBRA-OE mice, a nonsignificant increase in p-YAP/YAP expression was also observed, but there was no change in p-LATS/LATS expression (Supplemental Figure 2). The finding of increased p-YAP expression in podocytes of ADR-treated KIBRA-OE mice was supported by the demonstration of significantly reduced nuclear YAP immunofluorescent staining, with a predominance of cytoplasmic YAP staining in podocytes of ADR-treated KIBRA-OE mice compared with the podocytes of ADR-treated control mice (Figure 6). The results of both immunostainings shown in Figures 5 and 6 complement our in vitro findings of increased cytoplasmic YAP localization in KIBRA-OE human podocytes here (Figure 1) and as previously published in KIBRA-OE mouse podocytes (20). At least in part as a consequence of the loss of protective YAP-regulated signaling from podocyte nuclei, ADR-treated KIBRA-OE mice demonstrated significant reduction in podocyte number compared with ADR-treated control mice (Figure 7), thus linking KIBRA-induced YAP cytoplasmic localization with chronic glomerular injury in ADR-induced nephropathy.

### Glomerular WWC1 expression is associated with glomerular disease progression.

**Figure 8:**
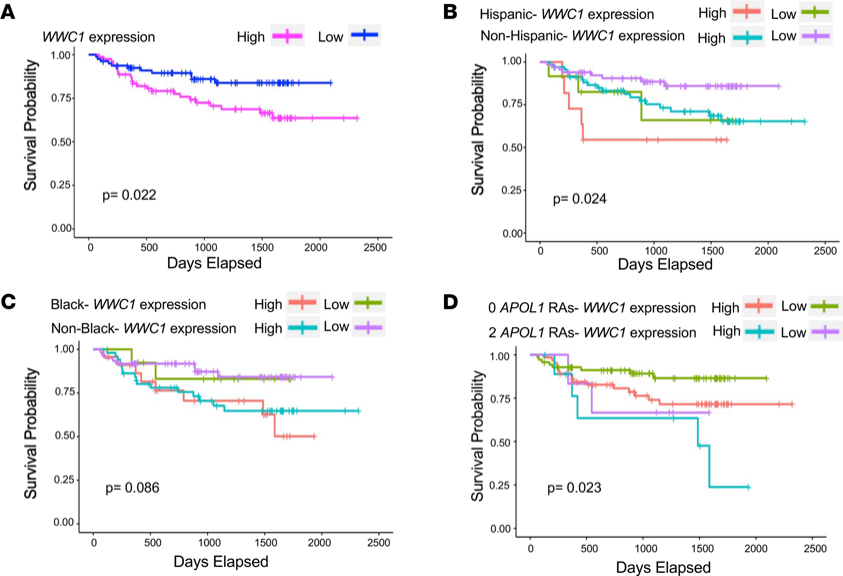
Glomerular disease outcomes correlate with *WWC1* mRNA expression. (**A**) Kaplan-Meier survival curves showing survival by glomerular *WWC1* expression levels. The bottom 50th percentile corresponds to *WWC1* expression lower than 4.203 (median value), and the top 50th percentile corresponds to *WWC1* expression greater than 4.203. Thirty-five out of 160 patients (21.9%) progressed to the composite endpoint of ESRD or 40% baseline eGFR reduction. (**B**) Kaplan-Meier survival curves showing survival stratified by glomerular *WWC1* expression levels and ethnicity (*n* = 159 patients, 1 patient with unknown ethnicity excluded). (**C**) Kaplan-Meier survival curves showing survival stratified by glomerular *WWC1* expression levels and race (*n* = 149 patients, 7 patients with mixed race and 4 patients with unknown race excluded). (**D**) Kaplan-Meier survival curves showing survival stratified by glomerular *WWC1* expression levels and *APOL1* genotype, defined as either 2 high-risk *APOL1* alleles (*G1/G1*, *G1/G2*, or *G2/G2*) or 0 high-risk *APOL1* alleles (*n* = 151 patients, 9 patients with 1 high-risk *APOL1* allele excluded). *P* values were calculated using the log-rank test. RAs, risk alleles.

Work by us and others has demonstrated that KIBRA expression is increased in human FSGS biopsy cases compared with normal kidney tissue (20). Here we tested the association between glomerular *WWC1* expression and longitudinal kidney disease outcomes. We analyzed a data set of glomerular transcriptomic profiles derived from kidney biopsies of patients from the NEPTUNE study, an NIH-funded multicenter collaborative consortium investigating the pathogenesis of primary glomerular diseases (29). Among 160 participants for whom both glomerular *WWC1* expression levels (determined by RNA sequencing) and renal survival data were available, 35 patients (21%) reached the composite outcome of either end-stage renal disease (ESRD) or at least 40% baseline estimated glomerular filtration rate (eGFR) decline at an average of 520 days. The baseline demographic and clinical characteristics of the NEPTUNE participants are outlined in Table 1. Increased glomerular *WWC1* expression levels were significantly associated with reduced renal survival (Figure 8A), suggesting the prognostic importance of *WWC1* upregulation to the progression of glomerular disease. Based on mean *WWC1* expression levels, the “high *WWC1* expression” subgroup (top 50th percentile) had an approximately 1.4-fold increase in glomerular *WWC1* expression levels over that of the “low *WWC1* expression” subgroup (bottom 50th percentile). Renal survival differed when patients were grouped by *WWC1* expression levels and ethnicity (Figure 8B), self-reported race (Figure 8C), and high-risk versus low-risk *APOL1* (which encodes Apolipoprotein L1) genotype, where high-risk was defined as 2 *APOL1* risk alleles (*G1/G1*, *G2/G2*, and *G1*/*G2*) and low-risk was defined as 0 *APOL1* risk alleles (Figure 8D). Further analyses revealed significantly worse renal survival with elevated glomerular *WWC1* expression in the specific subgroups of non-Hispanic and non-Black patients (Supplemental Figure 3, A and B) and decreased renal survival in the *APOL1* low-risk patients (Supplemental Figure 3C), though this difference was not statistically significant. Female patients with increased glomerular *WWC1* expression also showed a nonsignificant decrease in renal survival (Supplemental Figure 4A). Cross-sectional analysis at the time of renal biopsy revealed a nonsignificant correlation between glomerular *WWC1* expression and proteinuria (Supplemental Figure 4B) and no correlation between glomerular *WWC1* expression and level of kidney function (Supplemental Figure 4C), although the median eGFR of 86 mL/min for the cohort indicated milder disease.

In a Cox proportional hazards model, univariate analysis revealed that glomerular *WWC1* expression, evaluated as a continuous variable, was significantly associated with the composite renal outcome (HR 1.66, 95% CI 1.05–2.62, *P* = 0.03), as was Hispanic ethnicity (HR 2.53, 95% CI 1.09–5.88, *P* = 0.03) and eGFR at biopsy (HR 0.99, 95% CI 0.98–0.9995, *P* = 0.04). In the multivariable Cox regression analysis, the relationship between glomerular *WWC1* expression and renal survival remained significant after adjusting for ethnicity (Hispanic versus non-Hispanic) and eGFR at biopsy, as shown in Table 2. These findings overall suggest that *WWC1* may be an important risk gene for kidney disease progression among primary glomerulopathies.

## Discussion

We demonstrate for the first time to our knowledge that podocyte-specific KIBRA overexpression in vivo enhances susceptibility to acute and chronic podocyte injury and glomerular disease. We also show that increased *WWC1* glomerular expression correlates with decreased renal survival in biopsy-proven glomerular disease. We have built on our previously published findings to show and quantify KIBRA-induced disturbances of focal adhesion complexes in human podocytes. These data establish the connection between KIBRA-mediated regulation of the Hippo signaling pathway in podocytes and glomerular disease progression.

Our data suggest that these KIBRA-promoted effects are mediated to a large extent via YAP inhibition, given the findings of cytoplasmic YAP localization in KIBRA-OE human podocytes with a very apparent injury phenotype at baseline and of both increased phosphorylated YAP and reduced nuclear/increased cytoplasmic YAP in KIBRA-OE mice with heightened susceptibility to acute PS-induced FP effacement and ADR-induced FSGS. Additionally, they confirm the importance of the Hippo signaling pathway to the regulation of podocyte cytoarchitecture. Hippo pathway effectors YAP and TAZ have been identified as important cellular mechanosensors and mechanotransducers, in that their subcellular localization and transcriptional activity are altered in response to extracellular cues, including mechanical stretch, actin cytoskeletal changes, rigidity of the extracellular matrix, and cell density, among others (30, 31). YAP and TAZ are also involved in critical feedback loops with respect to assembly of actin fibers and focal adhesions (32, 33). RhoA, a member of the actin-regulating family of GTPases, has recently been shown to bind KIBRA/NF2 and deactivate Hippo signaling (34), and RhoA also indirectly promotes YAP nuclear shuttling through the formation of actin bundles and stress fibers in response to cell spreading (35, 36). Furthermore, it was recently demonstrated that YAP and TEAD promote the transcription of genes involved in focal adhesion assembly and that loss of nuclear YAP disrupts focal adhesion formation and cytoskeletal integrity in mesenchymal stem cells and various cancer cell lines, for example, thus impacting cell biomechanics and adhesivity (33, 37). These data also agree with our findings of increased cytoplasmic YAP expression with reduction of focal adhesions in KIBRA-OE human podocytes. Additionally, given published data showing that YAP can inactivate glycogen synthase kinase 3β (GSK3β) (38) and that loss of GSK3β ameliorates ADR-induced nephropathy with preservation of actin integrity and reduced activation of paxillin (39), it is possible that increased activity of GSK3β in the setting of KIBRA-induced YAP inhibition may also contribute to the significant injury susceptibility observed with KIBRA overexpression. Here, we focused on the localization, expression, and phosphorylation of YAP specifically over TAZ given that our group had previously showed that podocyte-specific YAP deletion in mice led to the development of a severe glomerular disease phenotype (22), while Chen et. al demonstrated a milder glomerular injury pattern in podocyte-specific TAZ-knockout mice (40).

It is possible that KIBRA may also regulate the podocyte actin cytoskeleton and focal adhesions in a more direct manner and independent of other Hippo pathway components. For example, the actin bundling protein synaptopodin is known to be a direct interacting partner of KIBRA (9), and we previously showed that KIBRA overexpression in mouse podocytes led to transcriptional downregulation of *Synpo* (20). The dysregulation of actin fibers could also be the result of the disturbance of a normal KIBRA/synaptopodin relationship, which warrants further investigation. KIBRA also directly interacts with the scaffolding protein angiomotin (Amot) (41), which was recently demonstrated to bind the focal adhesion–related protein talin and to promote actin filament formation at focal adhesions in endothelial cells (42). Another KIBRA-interacting partner, citron rho-interacting kinase (CIT kinase) (41), regulates actin dynamics during cytokinesis in HeLa cells (43). Finally, KIBRA was recently shown to serve as a scaffold for the exocyst-atypical protein kinase C (aPKC) complex formation that is crucial to migration of podocytes, and this complex also promotes the phosphorylation of paxillin via activation of JNK1 and ERK1/2 in NRK cells (44). Therefore, while crosstalk between the Hippo signaling pathway and key components of the podocyte cellular architecture is extensive, the direct relationship between KIBRA and non-Hippo regulators of the actin cytoskeleton and focal adhesions needs to be explored as well. KIBRA interactions with non-Hippo members could potentially account for the conflicting findings of protection conferred by KIBRA in podocytes that was noted by one study, though only limited supporting in vitro data with KIBRA silencing was presented there (45). The findings by our group (20, 22) and others (21, 46) largely support our central hypothesis that KIBRA is a mediator of podocyte injury and glomerular disease through activation of the Hippo pathway.

Our finding that podocyte-specific overexpression of KIBRA in mice was not sufficient to induce detectable podocyte injury or significantly alter phosphorylation of LATS or YAP, as opposed to KIBRA-OE podocytes with increased Hippo pathway activity and altered cell morphology at baseline, likely can be explained by the limitations imposed by cell culture model systems. Additionally, KIBRA protein expression was increased 7-fold in cultured overexpression cells, compared with the 2-fold increase observed in our transgenic mouse model. Another explanation is the potential 2-hit injury mechanism in vivo that has been proposed for several other forms of nephropathy, particularly in adults. One such example of this is APOL1-associated kidney disease. Though between 11% and 32% of patients of African ancestry demonstrate high-risk *APOL1* genotypes (47), the lifetime risk of developing chronic kidney disease for these carriers is approximately 20%, and recent GWAS studies show that *APOL1-*environment interactions may be more relevant to the development of APOL1-associated nephropathy than are *APOL1* interactions with other SNPs (48). In a similar manner, KIBRA expression and pathogenicity in vivo are likely modulated by environmental and genetic interactions. The upstream regulation of KIBRA expression remains an area for future study, although transcription factors TEAD4 and transcription factor 7–like 2 (TCF7L2) and YAP have been identified as potential positive regulators of *WWC1* expression (49). These findings introduce the interesting possibility of YAP-KIBRA feedback and support the preservation of nuclear/active YAP as a target for future podocyte-specific therapy development. Importantly, here the degree of KIBRA overexpression in our transgenic mice mirrors the increased *WWC1* expression seen in the NEPTUNE cohort (*WWC1* glomerular expression 1.4-fold greater in “high *WWC1*” versus “low *WWC1*” subgroups), indicating that our mouse model accurately reflects a physiologic increase in KIBRA expression.

Our data show a potential interaction between *APOL1* status and *WWC1* expression that could be the basis for further study. A limitation here is that the small number of NEPTUNE patients with 2 *APOL1* risk alleles and glomerular *WWC1* expression levels available (*n* = 16 patients) restricts any conclusion as to whether *WWC1* expression levels significantly increase the risk of renal disease for patients based on *APOL1* genotypes. Given the inclusion of patients in NEPTUNE with minimal change disease, membranous nephropathy, IgA nephropathy, and FSGS, this may suggest a potentially common Hippo-mediated injury pathway in podocytes that needs to be explored further in order to identify potential targets against which novel therapeutics may be leveraged in the future. However, due to limited patient sample size and low event rate of the composite renal endpoint, we were not able to assess differences between *WWC1* expression and disease outcomes based on glomerular disease subtype, though this is an important consideration for future analyses.

In summary, our results demonstrate the importance of KIBRA as a regulator of podocyte focal adhesion dynamics whose overexpression enhances susceptibility to acute and chronic glomerular injury and correlates with decreased renal survival in patients with biopsy-proven glomerular disease.

## Methods

### Overexpression of KIBRA in human podocytes.

pBabe-puro and pBabe-puro-Kibra were purchased from Addgene. The pBabe-puro plasmids along with the helper plasmids pUMVC and VSVG were transfected into HEK293 cells at 70% confluence using FuGENE 6 (Promega) to generate viral supernatant. Wild-type undifferentiated human podocytes (a gift from Moin Saleem, University of Bristol, Bristol, United Kingdom) (50) were infected for 24 hours and then selected. Noninfected podocytes were removed by selection in 2 μg/mL puromycin (Sigma-Aldrich). Podocytes were selected for 1 week, after which 1 μg/mL puromycin was used as the maintenance dose.

### Western blot analysis.

Human podocyte pellets were lysed in RIPA buffer and Western blotting was performed according to standard protocols. Each lane contained approximately 30 μg of total protein. Density for the protein of interest was normalized to GAPDH using ImageJ software (NIH). Blots were probed with an anti-KIBRA antibody (Cell Signaling Technology, 8774). This experiment was repeated 4 times.

### Immunofluorescence.

Following 10 days of differentiation at 37°C, human podocytes were fixed on coverslips with 4% PFA/4% sucrose in PBS. Prior to staining, cells were permeabilized with 0.3% Triton X-100 in PBS, and then incubated for 30 minutes with blocking serum (2% fetal calf serum, 2% bovine serum albumin, 0.2% fish gelatin) and then incubated for 1 hour with primary antibodies against paxillin (BD Biosciences, 610619) and YAP (Novus Biologicals, NB110-58358). The sections were then washed and incubated for 1 hour with the following secondary antibodies conjugated with fluorochromes: Alexa Fluor 488 goat anti–rabbit IgG (Thermo Fisher Scientific, A-11008) and Alexa Fluor 555 goat anti–mouse IgG (Thermo Fisher Scientific, A32723). Rhodamine-phalloidin (Thermo Fisher Scientific, R415) was used to stain actin fibers and DAPI was used as a nuclear counterstain. Immunostained kidney sections were mounted with Mowiol mounting medium (Sigma-Aldrich, 81381).

A similar protocol was followed for immunofluorescent staining of frozen kidney tissue sections from ADR-treated control and KIBRA-OE mice. After incubation with the same blocking serum above, tissue sections were incubated for 1 hour with primary antibodies against YAP (Novus Biologicals, NB110-58358) and synaptopodin (R&D Systems, MAB8977). The sections were then washed and incubated for 1 hour with the following secondary antibodies conjugated with fluorochromes: Alexa Fluor 488 goat anti–rabbit IgG (Thermo Fisher Scientific, A11008) and Alexa Fluor 555 goat anti–mouse IgG (Thermo Fisher Scientific, A32727). DAPI was used as a nuclear counterstain. Confocal microscopy was performed using a Leica SP5 DMI microscope.

### TIRF microscopy.

TIRF microscopy was performed as previously described to obtain a detailed assessment of focal adhesion parameters using a Leica DMi8 Infinity TIRF microscope and LASX (v.3.6) (51). Focal adhesion morphometrics were assessed using immunofluorescent staining of paxillin (BD Biosciences, 610619), with an evanescent field depth of 90 nm, imaged under PBS using a 1.4 NA Leica 63× oil TIRF objective at 30°C. To determine per-cell morphometric values, simultaneous widefield images of the actin cytoskeleton and nuclei were obtained using rhodamine-phalloidin (Thermo Fisher Scientific, R415) and DAPI, respectively. All images were systematically processed in an unbiased and blinded manner. Cells and focal adhesions were segmented using Cellpose (52) on the phalloidin and paxillin channels, respectively. For cellular segmentation, a subset of randomly selected images was chosen and manually segmented. A new Cellpose model was trained with these manually segmented images starting from the built-in “cyto2” model. For focal adhesion segmentation, a subset of randomly chosen segmented cells was chosen and the focal adhesions were manually segmented. A new Cellpose model was then trained using these manually segmented focal adhesions.

### Establishment of inducible KIBRA-OE mice.

*WWC1* cDNA was subcloned into the pTRE3G vector (Takara Bio). The transgene was digested to remove the vector backbone. Pronuclear injections with the *TRE/WWC1* transgene were done in FVB/N oocytes, generating *TRE-WWC1* mice. To identify founders, we designed primers to span the *TRE* promoter and *WWC1* (5′-CTGGAGCAATTCCACAACAC-3′ and 5′-AGTCAGCGAAGGTGAGTGGT-3′). These mice were then crossed with *Nphs1-rtTA* mice, also in a FVB/N background (a gift from Jeffrey Miner, Washington University School of Medicine, St. Louis, Missouri, USA) to generate Dox-inducible podocyte-specific KIBRA-OE mice. The genetic background of mice was confirmed as FVB/N by SNP analysis (DartMouse Speed Congenic Core Facility at the Geisel School of Medicine at Dartmouth).

The podocyte-specific expression of KIBRA protein was induced by the administration of Dox-supplemented chow (2000 mg/kg, Envigo). Overexpression of KIBRA was verified by IHC staining of paraffin-embedded kidney sections following sacrifice, kidney harvesting, and fixation in 3% PFA.

### IHC.

Kidney sections that had been fixed with 3% PFA and embedded in paraffin were deparaffinized, and endogenous peroxidase was inactivated with H_2_O_2_. Sections were then blocked in 2% goat serum in PBS for 1 hour at room temperature and then incubated with the primary antibody at 4°C overnight. The following day, sections were washed 3 times with PBS and then incubated with the secondary antibody for 30 minutes. Positive staining was revealed by peroxidase-labeled streptavidin and diaminobenzidine substrate, with a fixed exposure time per antibody for all experiments. The negative control included a section stained with only secondary antibody. Antibodies against the following proteins were used in this study: KIBRA (Novus Biologicals, NBP1-92052), p-LATS 1/2-Thr-1079/Thr-1041 (Abcam, ab111344), LATS1 (Proteintech, 17049-1-AP), p-YAP-Ser-127 (Cell Signaling Technology, 13008), and YAP (Novus Biologicals, NB110-58358).

### Quantification of immunostaining.

IHC images were obtained using a Hamamatsu NanoZoomer S210 digital slide scanner. Fiji ImageJ software was used to measure the level of immunostaining for KIBRA in glomeruli. Color deconvolution was selected and specified to DAB vectors. Images were converted to black-and-white and a threshold level was selected for each antibody and used for each image quantified. Glomerular regions were selected for measurement of percentage area with positive staining based on the set threshold level for each antibody. At least 35 glomeruli were selected and quantified per mouse.

Immunofluorescence images were obtained using a Leica SP5 DMI microscope. Imaris software was used to measure the level of immunostaining for YAP. Surfaces were created for DAPI- and synaptopodin-labeled areas, and a threshold value for YAP staining was selected, whereby mean intensity measured above this threshold was considered “positive” and below was considered “negative” for YAP staining. The mean intensity of YAP staining within DAPI-designated surfaces colabeled for synaptopodin on the periphery of each glomerulus was also quantified, representing nuclear YAP expression in podocytes. At least 5–6 glomeruli were quantified per mouse and a total of 4 mice were quantified per group (ADR-treated controls and KIBRA-OE mice).

### Mouse kidney histologic grading.

Kidney samples were fixed in 3% PFA, embedded in paraffin, sectioned to 4 μm thickness, and then stained with periodic acid–Schiff (PAS). PAS-stained kidney sections were then used for kidney histology. Histological scoring was performed in a blinded manner by the renal pathologist. Glomerulosclerosis was graded on a semiquantitative scale (0 to 4+), as follows: 0 (none), 1+ (involving 1%–10% of all glomeruli sampled), 2+ (involving 11%–25% of glomeruli), 3+ (involving 26%-50% of glomeruli), and 4+ (involving >50% of glomeruli). An average of 25 glomeruli sampled per animal were evaluated.

### PS model.

Ten-week-old KIBRA-OE (*n* = 6, 3 female and 3 male mice) and control (*n* = 5 *Nphs1-rtTA^–^* *TRE-WWC1^+^*, 2 female and 3 male mice) mice received Dox chow for 2 weeks mice to induce KIBRA overexpression and then were anesthetized with ketamine/xylazine, and their kidneys were perfused with 2 mg/mL PS in HBSS in situ through the renal artery at an infusion rate of 7.5 mL/min for 22 minutes, followed by 50 mL of 3% PFA, as previously described (20). Kidneys were harvested following perfusion, sectioned transversely into thirds, and fixed in a solution of 2.5% glutaraldehyde at 4°C for TEM.

### TEM and FP quantification.

TEM images of kidney sections fixed in 2.5% glutaraldehyde at 4°C were obtained on a Hitachi HT7000 transmission electron microscope. Images at ×3000 magnification were used for FP quantification, while representative higher-power images were obtained at ×10,000 magnification. ImageJ software (NIH) was used to measure the length of the peripheral GBM, and the number of FPs per GBM length were counted by a blinded observer. This was done for at least 3 glomeruli per mouse, with at least 10–15 images obtained per glomerulus.

### ADR nephropathy model.

KIBRA-OE (*n* = 13, 6 female, 7 male mice) and control (*n* = 9 *Nphs1-rtTA^+^* *TRE-WWC1^–^* or wild-type, 4 female, 5 male mice) 9.5-week-old mice were fed Dox chow for 1 week to induce KIBRA expression and then received retro-orbital injections of either ADR (15 mg/kg; ADR group) or PBS (15 mg/kg; PBS group). Dox chow was continued following ADR injections until the time of sacrifice at 6 weeks after ADR injection. Weights were monitored daily for the first week following injection, and then weekly for an additional 5 weeks until the day of sacrifice. Urine was collected weekly.

### Proteinuria quantification.

Screening for albuminuria was performed using a 10% SDS–polyacrylamide gel followed by Coomassie blue staining. Bovine serum albumin standards (1 μL of 0.1, 0.5, 1, and 5 μg/μL) and 5 μL of urine was used from each mouse were used to qualitatively assess the presence of albuminuria.

Urine albumin was determined using a commercial assay from Bethyl Laboratory Inc. Urine creatinine was quantified using commercial kits from Cayman Chemicals. Urine albumin excretion is expressed as the ACR (mg/g).

### Quantification of podocyte number.

Podocyte number per glomerulus for all ADR-treated mice (*n* = 9 control mice, 13 KIBRA-OE mice) was quantified using the Weibel-Gomez method. One-micrometer-thick sections from blocks processed for TEM were stained with toluidine blue and observed with a light microscope. Twenty glomeruli per kidney were imaged. A grid of points was superimposed over each image and the number of grid points falling on podocyte nuclei and the number of points falling on the glomerular profiles were counted. Also, the number of podocyte nuclei was counted. It was assumed there was only 1 nucleus per podocyte. The sums of these 3 parameters over the 20 glomeruli per kidney were used to determine the average podocyte numerical density and average glomerular volume per kidney. The product of these 2 values is the average podocyte number per kidney (Figure 7B) (53, 54).

### KIBRA expression and association with outcome in human glomerular disease.

The NEPTUNE cohort (ClinicalTrials.gov NCT01209000) is a multiprospective observational cohort of children and adults enrolled at the time of first clinically indicated biopsy and followed for up to 5 years (29). Detailed phenotypic information, including pathology diagnosis, demographics, medical history, laboratory results, blood and urine samples for measurement of serum creatinine and urine protein/creatinine ratio (UPCR) were collected at each study visit. eGFR (mL/min/1.73 m^2^) was calculated using the CKD-Epi formula for participants 18 years of age or older old and the modified CKiD-Schwartz formula for participants less than 18 years old, with an average taken for adolescents (55–57). Loss of kidney function is defined as a combination of ESRD (initiation of dialysis, receipt of kidney transplant or eGFR < 15 mL/min/1.73 m^2^ measured at 2 sequential visits) or 40% reduction in eGFR from the baseline visit. Tissue obtained at the time of biopsy was manually microdissected to separate the glomerular from the tubulointerstitial compartment and genome-wide RNA sequencing was performed by the University of Michigan Advanced Genomics Core (https://brcf.medicine.umich.edu/cores/advanced-genomics/). Gene transcript level is reported as fragments per kilobase per million mapped reads (FPKM).

### Statistics.

Data for Figures 1–7 are expressed as mean ± SD. For imaging and Western blot analysis, normality was determined using the Shapiro-Wilk test and a *P* value of 0.05 or greater. If the distribution was normal, an unpaired 2-tailed *t* test was performed, where *P* less than 0.05 was considered statistically significant. If the variances between groups were significantly different, an unpaired 2-tailed *t* test with Welch’s correction was performed, where *P* less than 0.05 was considered statistically significant. For data sets with non-normal distribution, the Wilcoxon-Mann-Whitney rank-sum test with a nonparametric 95% CI was used. Statistical analyses were performed using GraphPad Prism version 9.3.1.

Kaplan-Meier survival analysis was performed in R using the packages survival and survminer and survival curves were compared using the log-rank test. A Cox regression analysis was performed to assess variables associated with a 40% decline in eGFR or ESRD. Univariate variables that were significantly associated with the outcome of interest (*P* < 0.1) were utilized in the multivariable Cox regression model. Statistical analyses were performed using R version 4.1.3 (https://www.r-project.org).

### Study approval.

All animal procedures were performed according to protocols approved by the IACUC at the Icahn School of Medicine at Mount Sinai.

## Author contributions

KM, YY, MDC, ERB, CM, JH, JRL, FS, LHM, REG, JMB, HHW, JF, EUA, JCH, JSW, and KNC performed the experiments, data acquisition, and data analysis. KM, JSW, and KNC conceptualized and designed the studies. All authors participated in the drafting and critical appraisal of the manuscript. They all approve of the final version and agree to be accountable for all aspects of the work.

## Supplementary Material

Supplemental data

## Figures and Tables

**Table 1:**
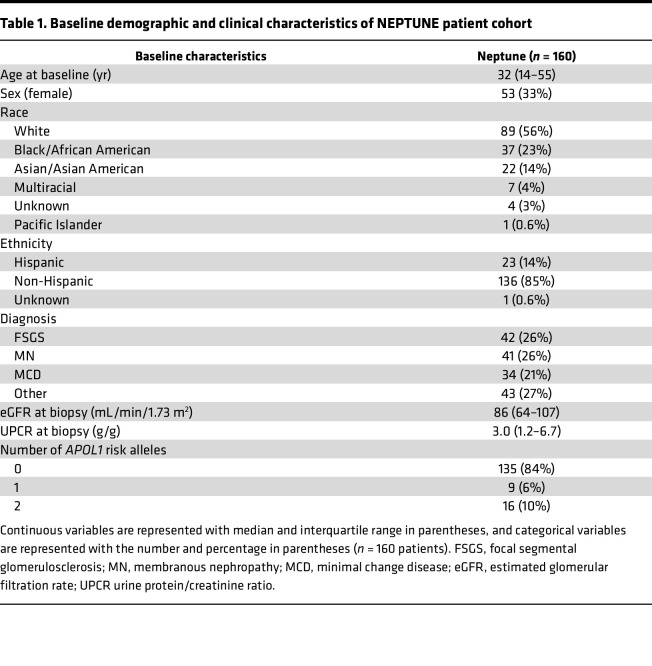
Baseline demographic and clinical characteristics of NEPTUNE patient cohort

**Table 2:**
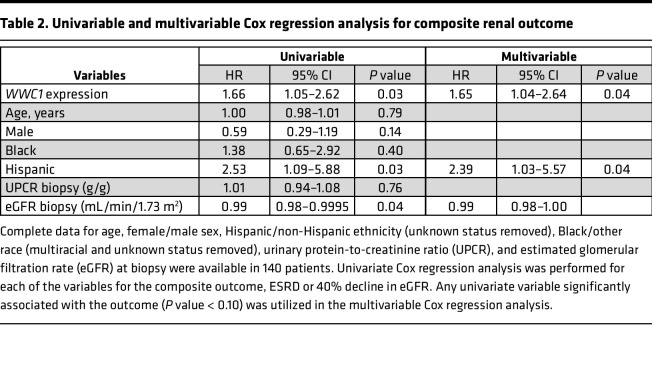
Univariable and multivariable Cox regression analysis for composite renal outcome

## Version 1 02/28/2023

In-Press Preview

## Version 2 04/10/2023

Electronic publication
