# Supplementary material for: KIBRA upregulation increases susceptibility to podocyte injury and glomerular disease progression
Source: JCI Insight. 2023 Apr 10;8(7):e165002. doi: 10.1172/jci.insight.165002 (PMC10132156; doi:10.1172/jci.insight.165002)
Supplement: Supplemental data [file jciinsight-8-165002-s011.pdf]

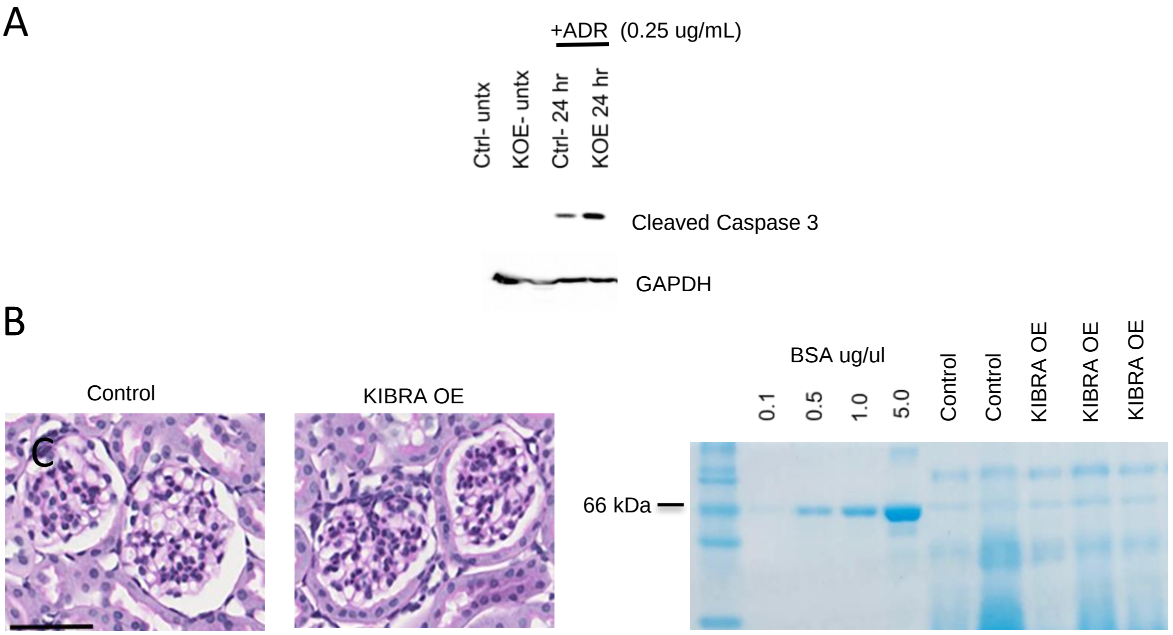

Supplemental Figure 1. Apoptosis in KIBRA overexpression podocytes and baseline histology of KIBRA OE mice. (A) Increased apoptosis in adriamycin-treated KIBRA OE podocytes, as assessed by WB for cleaved caspase 3 (n= 3 experiments). ADR, adriamycin. (B) KIBRA OE mice show normal histology and lack albuminuria (n=2 control, 3 KIBRA OE mice). Scale bar= 50 µm.

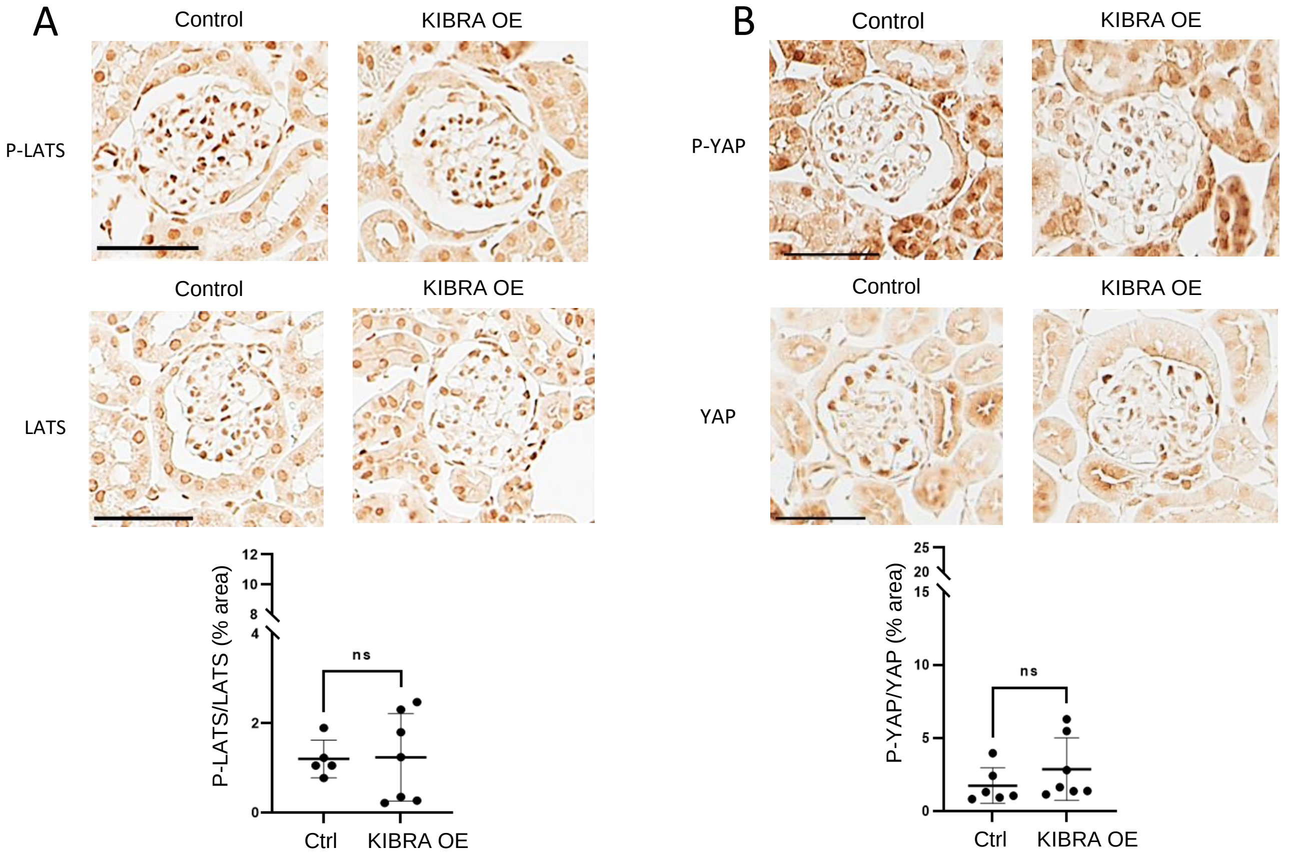

Supplemental Figure 2. Hippo pathway signaling in PBS-treated KIBRA OE mice. PBS-treated KIBRA OE mice show (A) similar P-LATS/LATS expression (n= 5 control, 7 KIBRA OE mice) and (B) a non-significant increase in P-YAP/YAP expression (n= 6 control, 7 KIBRA OE mice). Original magnification 40x. Scale bar= 50 μm. ns, not significant; two-tailed t test (P-LATS/LATS), two-tailed Mann-Whitney test (P-YAP/YAP).

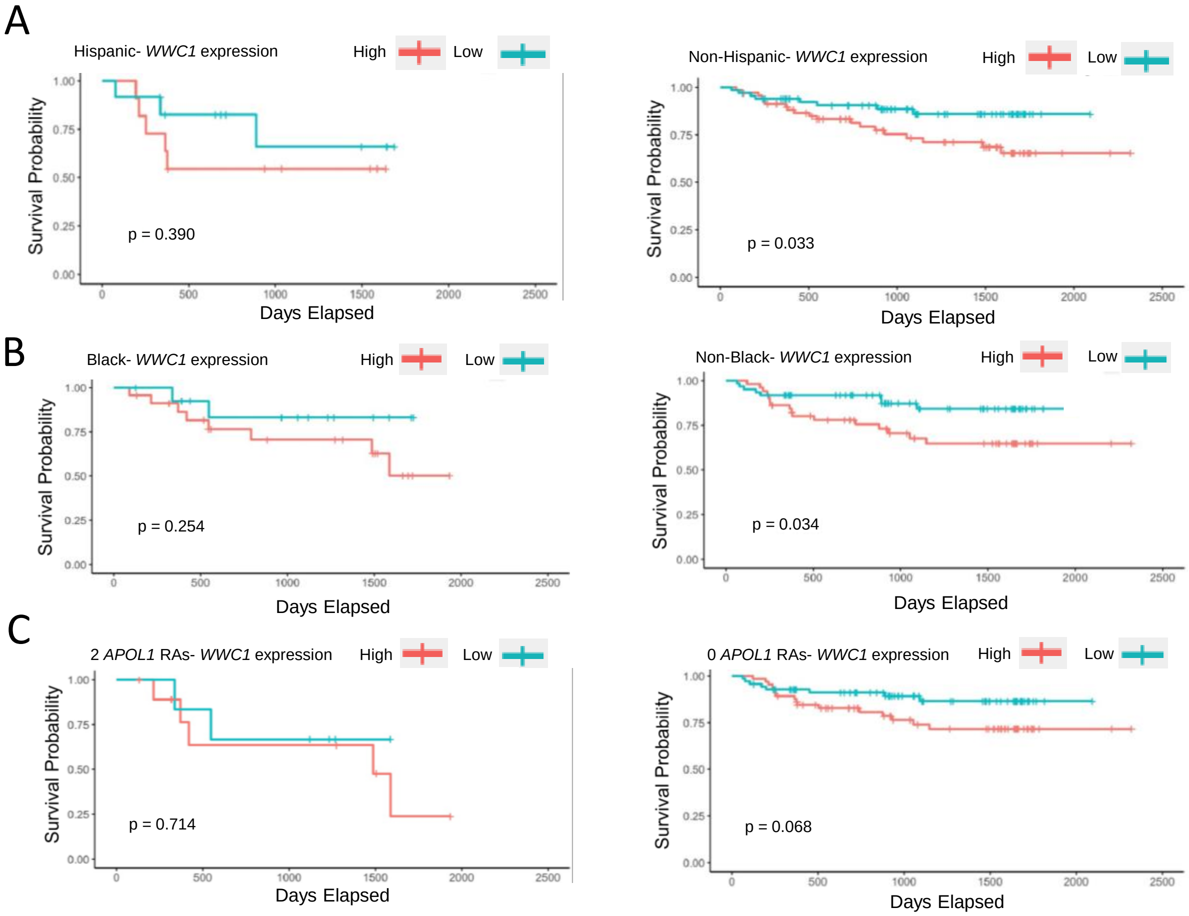

Supplemental Figure 3. Subgroup analysis of glomerular *WWC1* expression and renal survival. (A) Kaplan-Meier survival curves showing survival stratified by glomerular *WWC1* expression levels and Hispanic ethnicity (n=23 patients) and non-Hispanic ethnicity (n=136 patients). (B) Kaplan-Meier survival curves showing survival stratified by glomerular *WWC1* expression levels and Black race (n= 37 patients) and non-Black race (n=112 patients). (C) Kaplan-Meier survival curves showing survival stratified by glomerular *WWC1* expression levels and *APOL1* status defined as either the presence of 2 high-risk *APOL1* alleles (*G1/G1, G1/G2,* or *G2/G2*; n= 16 patients) or 0 high-risk *APOL1* alleles (n= 135 patients). The top 50th percentile correspond to *WWC1* expression greater than 4.203 (median value). P-values were calculated using the log rank test. RAs, risk alleles.

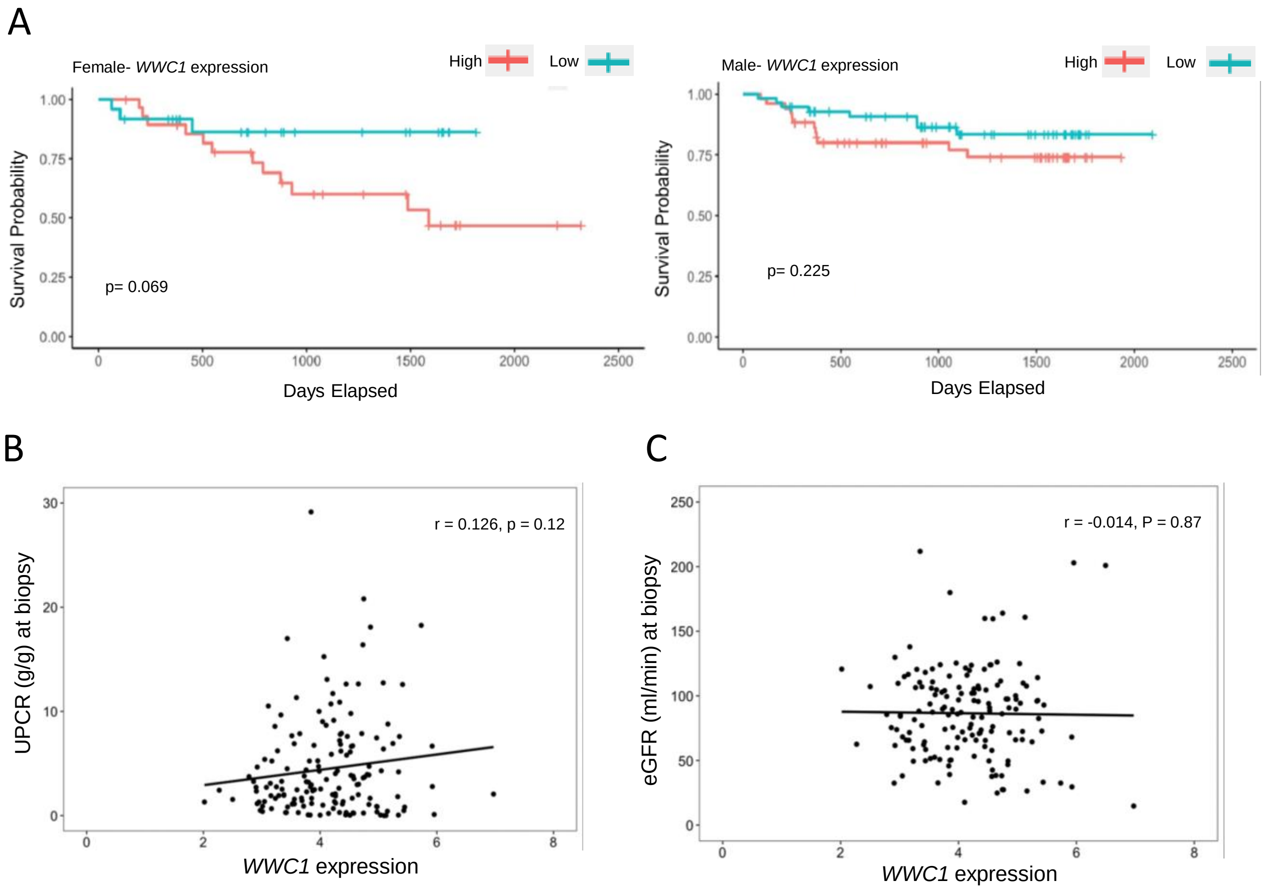

Supplemental Figure 4. Longitudinal subgroup analysis and cross-sectional analysis of glomerular *WWC1* expression. (A) Kaplan-Meier survival curves showing survival stratified by glomerular *WWC1* expression levels and female sex (n=53 patients) and male sex (n=107 patients). The top 50th percentile correspond to *WWC1* expression greater than 4.203 (median value). P-values were calculated using the log rank test. (B) Pearson's correlation between glomerular *WWC1* expression and urine protein-to-creatinine ratio (UPCR) at the time of kidney biopsy (n= 157 patients). (C) Pearson's correlation between glomerular *WWC1* expression and estimated GFR (eGFR) at the time of kidney biopsy (n= 154 patients).
